# Correlations between Retinal Arterial Morphometric Parameters and Neurodegeneration in Patients with Type 2 Diabetes Mellitus with No or Mild Diabetic Retinopathy

**DOI:** 10.3390/medicina57030244

**Published:** 2021-03-05

**Authors:** Ioana Damian, Simona Delia Nicoară

**Affiliations:** 1 Department of Ophthalmology, “Iuliu Hațieganu” University of Medicine and Pharmacy, 8 V. Babes str., 400012 Cluj-Napoca, Romania; ioana.damian@umfcluj.ro; 2 Medical Doctoral School, University of Oradea, 1 Universitatii Str., 410087 Oradea, Romania; 3 Department of Ophthalmology, Emergency County Hospital Cluj, 3–5 Clinicilor Str., 400006 Cluj-Napoca, Romania

**Keywords:** diabetic retinopathy, diabetic neuropathy, neurodegeneration, optical coherence tomography, densitometry, GCL thickness, RNFL thickness, retinal arterial wall thickness, wall-to-lumen-ratio, wall cross-sectional area

## Abstract

*Background and Objectives*: In patients with diabetes mellitus (DM), the neural retina is starting to degenerate before the development of vascular lesions. Our purpose was to investigate the correlation between the retinal arterial morphometric parameters and structural neurodegeneration in patients with type 2 DM with no or mild diabetic retinopathy (DR). *Materials and Methods*: This is a prospective study including 53 eyes of patients with type 2 DM and 32 eyes of healthy controls. Based on SD-OCT (spectral domain—optical coherence tomography) images, using a micro-densitometry method, we measured the outer and luminal diameter of retinal arteries and calculated the AWT (arterial wall thickness), WLR (wall-to-lumen ratio), and WCSA (wall cross-sectional area). GCL (ganglion cell layer) and RNFL (retinal nerve fiber layer) thickness were analyzed in correlation with the retinal arterial morphometric parameters mentioned above. *Results*: GCL was thinner in the inner quadrants in the NDR (no DR) group compared to controls (*p* < 0.05). RAOD (retinal artery outer diameter), RALD (retinal artery lumen diameter), AWT, WLR, and WCSA were similar between groups. A regression model considering age, gender, duration of DM, and HbA1C was carried out. Central GCL thickness was correlated positively with RAOD (coefficient 0.360 per µm, *p* = 0.011), RALD (coefficient 0.283 per µm, *p* = 0.050), AWT (coefficient 0.304 per µm, *p* = 0.029), and WCSA (coefficient 3.90 per µm, *p* = 0.005). Duration of DM was positively correlated with WCSA (coefficient 0.311 per one year duration of diabetes, *p* = 0.043). *Conclusions*: Significant GCL thinning in the inner quadrants preceded the morphological retinal arterial morphometric changes, supporting the neurodegeneration as primary pathogenic mechanism in DR.

## 1. Introduction

It is estimated that 383 million people have diabetes mellitus (DM) worldwide and this number is expected to increase to 592 million by 2035 [1] and 693 million by 2045 [2]. Poorly controlled or untreated DM leads to diabetic retinopathy (DR), which is the leading cause of preventable visual impairment within the working-age population group in developing countries [3,4]. One in three people with DM has DR, which seems to be associated with an increased risk of life-threatening systemic vascular complications such as stroke, coronary heart disease, and heart failure [5].

Until recently, DR was considered to have an exclusively vascular pathogenesis [6]. The latest findings suggest that neurodegeneration plays an important role in the complex pathogenesis of DR involving various factors and mechanisms, such as hyperglycemia, dysregulation of growth factors, neurotrophic factors, chemokines, vasoactive agents, inflammatory, and adhesion molecules [1,3,7]. According to one theory, the neuronal and vascular elements respond independently to alterations induced by high glucose and only in late stages involving retinal detachment, neuronal function and survival are endangered. However, since neuronal, vascular, and glial cells are intimately connected into the neurovascular unit, this hypothesis seems unlikely. Another theory postulates that the neurons which are the most fragile and demanding cellular elements in the retina are the first to be affected by microenvironmental changes [3]. The American Diabetes Association defined DR as a tissue-specific neurovascular complication, which involves progressive disruption of the interdependence between multiple cell types in the retina [4]. One of the mechanisms seems to involve vascular endothelial growth factor (VEGF), which has a neuroprotective role, being released by the retina in the early phases of the disease in response to neuronal stress [8]. For example, ganglion-cell derived VEGF plays an important role as an autocrine-paracrine neurotrophic factor for neuron cell survival under stress condition [9]. Glutamate, which is known for its important role in retinal neuronal death, seems to upregulate VEGF production. In the initial phase VEGF acts as a pro-survival factor, but a prolonged upregulation leads to microvascular lesions [3,10]. Other mechanisms involved in neurodegeneration are the induction of oxidative stress by the aberrant production of mitochondria-derived reactive oxygen species (ROS) and the reduction of neuroprotective factors synthesized by the retina such aspigment epithelial-derived factor (PEDF), somatostatin (SST), and interstitial retinol-binding protein [11]. Moreover, Hernández et al. emphasized the role of advanced glycation end-products (AGEs), endoplasmic reticulum (ER) stress, and neuroinflammation in neuronal damage [12]. Inside the retinal neurovascular unit, neurons are receiving oxygen and energy, neurotransmitters are recycled, and waste is removed, while neuronal activity evokes localized reactions in large and small vessels, such as vasodilatation and increased blood flow to meet the energy demands of neuronal signal transduction and transmission [13,14].

The presence of neurodegeneration in DR was demonstrated morphologically by using SD-OCT (spectral domain—optical coherence tomography) and swept source optical coherence tomography (SS-OCT) that highlighted structural changes like reduction of the retinal nerve fiber layer (RNFL), ganglion cell layer (GCL) and inner plexiform layer, and by studies involving diabetic animal models in which apoptotic death of retinal ganglion cells and amacrine cells was demonstrated [12,13]. Functional deficits were proved using methods such as mf-ERG (multifocal electroretinography) where reduced electrical response was found [14] predominantly in the inner retina [13], standard automated perimetry, frequency doubling perimetry, or microperimetry [12]. Patients with DM without clinically overt microvascular changes presented the aforementioned changes, suggesting that neural retina is starting to degenerate before the development of vascular changes [13].

Retinal image analysis techniques such as SD-OCT, confocal adaptive optics scanning ophthalmoscope (AOSLO) together with OCT-angiography have enabled researchers to assess and analyze several retinal vessels parameters [12,15]. Most of the previous studies used retinal photography to measure vessel diameter manually or automatically, but this method only yields the retinal vessel caliber diameter without being able to provide any additional morphometric parameter, such as WCSA (wall cross-sectional area) or AWT (arterial wall thickness). Other research groups used SD-OCT images for measuring vascular diameter by A-scans at an axial high-resolution of 7 µm using either manual methods such as built-in SD-OCT software or ImageJ software, or an automatized method [16].

The micro-densitometry approach was used in studies to quantitatively assess the retinal width in a digital fundus or OCT image, the width of the vessel being estimated at half of the peak height of the intensity profile curve, providing a fast and robust estimation of the vessel’s edge [17].

In the current study we analyzed Spectralis SD-OCT images and measured retinal artery vessel diameter using a micro-densitometry method, i.e., full-width half-maximum (FWHM), to determine if there are any differences between the DM groups and control regarding retinal arteriolar parameters, such as diameter, AWT, WLR (wall-to-lumen ratio), or WCSA in patients with DM, and no or mild clinically detectable microvascular DR. We further analyzed the structural neurodegeneration in the groups by comparing GCL and RNFL thickness. Following these analyses, we investigated the possible correlations between retinal neurodegeneration and retinal artery vessel morphometric parameters in patients with no or mild DR.

## 2. Materials and Methods

### 2.1. Study Subjects

This prospective, single-center, comparative study adhered to the tenets of The Declaration of Helsinki and the protocol was approved by the Ethics Committee belonging to, Iuliu Hațieganu” University of Medicine and Pharmacy (IHUMP), Cluj-Napoca, Romania.

We included in the study 53 eyes belonging to patients with type 2 DM and 32 eyes of healthy controls. All participants were recruited between July 2018 and July 2019 at the Department of Ophthalmology, Emergency County Hospital Cluj, Romania. We divided the subjects into three groups: group 1, healthy subjects with no DM (33 eyes); group 2, subjects with type 2 DM and no DR (NDR) (32 eyes); and group 3, subjects with type 2 DM and mild non-proliferative DR (NPDR) (21 eyes). Inclusion criteria for the patients with DM were: (1) diagnosis of type 2DM for more than three months, and (2) diagnosis of no DR or mild NPDR. Exclusion criteria were: (1) DR in more advanced stages than mild NPDR; (2) diabetic macular edema (DME); (3) media opacities that precluded appropriate OCT imaging; (4) presence of other retinal diseases such as age-related macular degeneration, vitreo-macular traction, macular hole, epiretinal membrane, retinal vein occlusion, or macular scars; (5) glaucoma, optic neuropathies, or neurodegenerative diseases; (6) history of ocular trauma; (7) refractive errors of more than ±6 diopters (spherical equivalent); and (8) previous ocular surgery, focal or panphotocoagulation laser, anti-VEGF intravitreal injection. Healthy subjects were recruited from patients scheduled for routine ocular examination at the Department of Ophthalmology, Emergency County Hospital Cluj, Romania.

All subjects underwent ocular examination including best corrected visual acuity (BCVA) evaluation with Snellen chart, slit-lamp biomicroscopy of the anterior and posterior segment, contact tonometry, and OCT. The eye with the best visual acuitywas chosenfor theanalysis. If both eyes had the same BCVA, the eye with the highest OCT image quality was included in the study.

Written informed consent was obtained from the patients after explaining the required procedures and the details of the study.

ClinicalTrials.gov NCT04637217; https://www.clinicaltrials.gov/ct2/show/NCT04637217; accessed on 19 November 2020. 

### 2.2. SD-OCT Data Acquisition

All study subjects were imaged using Spectralis OCT (Heidelberg Engineering, Inc., Heidelberg, Germany).

All scans were acquired after pupil dilatation with 0.5% Tropicamide and 10% Phenylephrine. Images were obtained using the automated eye alignment eye tracking software (TruTrack; Heidelberg Engineering). The Spectralis “Posterior Pole” scanning protocol was used: Scanning area 30° × 25°, 61 horizontal raster linescentered on the fovea, to obtain volumetric retinal scans. The inbuilt Spectralis mapping software was used to perform measurements from each SD-OCT scan. Segmentation was automatically performed using the Spectralis software version 6.0 to obtain the following thickness measurements: central macular thickness (CMT), RNFL, GCL. We extracted values for the central 1, 3, and 6 mm ETDRS macular maps concentric ring. For the peripapillary RNFL all patients underwent circular OCT-scans around the ONH (optic nerve head) using Spectralis OCT + HRA (Heidelberg Engineering, Heidelberg, Germany) during the same visit. The scanning protocol consisted of a circular scan with 3.4 mm diameter centered on the ONH at the intersection of zone A with zone B. Simultaneously, near-infrared reflectance (IR) pictures were obtained. Only high-quality scans, which we defined asscans with a signal strengthof more than 25 dB (ranging from 0 = poor to 40 = excellent) and with individual retinal layers that could be identified were used for the analysis. All scans were performed by the same experienced operator. The signal strength for peripapillary RNFL scans was 28.94 dB in the control, 28.78 dB in the NDR group and 28.67 dB in the NPDR group.

### 2.3. Image Analysis Using FWHM Micro-Densitometry Method

**Figure 1:**
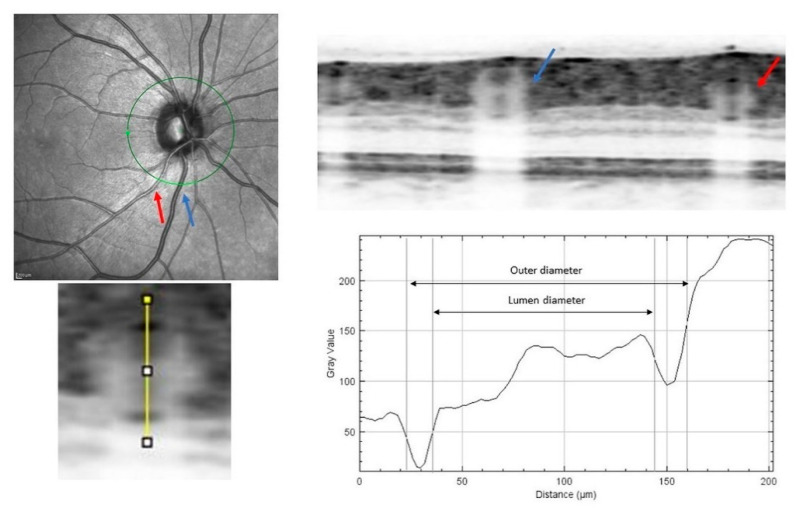
Measurement of the retinal vessel outer and lumen diameters in an OCT image using the micro-densitometry method. (Top left) A circular scan was performed across the vessels at the intersection of zone A to zone B. (Top right) The cross-sectional structure of the retinal artery (red arrow) and vein (blue arrow) could be identified in the OCT image. (Bottom left) The line selection vertically crossed the middle of the upper and lower vessel walls to produce an intensity profile. (Bottom right) The boundary points were estimated at half maximum intensity for each side of the two parabolas in the profile. The distance between the boundary points was calculated for the vessel outer and lumen diameters.

Before retinal vessels diameter measurement, the vertical-to-horizontal ratio of the OCT image was changed to 1:1 µm, and the image was magnified to the maximum (800%). For the FWHM-based measurement, magnified OCT image showing the retinal artery vessel of interest was saved as PNG file. The saved image was exported to ImageJ (software version 1.53a, National Institutes of Health, Bethesda, Maryland, USA https://imagej.nih.gov/ij/; accessed on 10 November 2020) [17]. The arteries were differentiated from the veins on infra-red images, based on anatomical characteristics of the two vessels (e.g., brightness, central reflex, and shape) and clinical experience of the grader. We measured the pixel length of 200 µm as given in the scale at the bottom of the OCT scan image, using the line tool. The intensity profile along a line vertically crossing the middle of the vessel was measured using the software (see Figure 1). On the intensity profile, the two parabolas that are opening upward represent the upper and the lower vessel walls in the OCT image. After determining the edge of the vessel arterial wall, as previously described [18,19], the outer and luminal diameters were measured. For every eye, the largest four retinal arteries were measured.

### 2.4. Retinal Artery Morphometry

**Figure 2:**
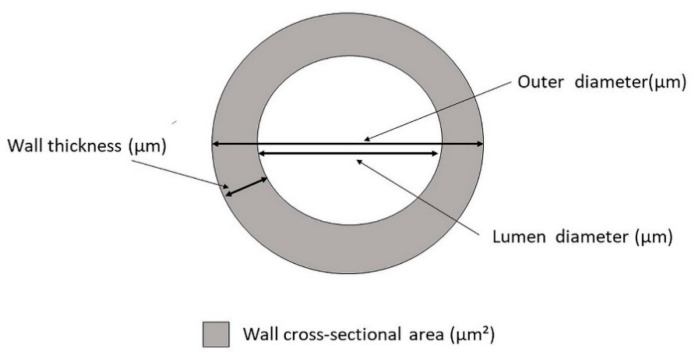
Cross-section through a simplified artery to illustrate retinal artery morphometric parameters.

In order to increase the precision, every outer and luminal diameter for each vessel was measured twice, the values were then averaged and further used in the study. Based on outer and lumen diameter, the following parameters were calculated: AWT (see Equation (1)), WLR (see Equation (2)), and WCSA (see Equation (3)) (see Figure 2)

(1) $$AWT=\frac{outer\;diameter-lumen\;diameter}{2}$$

(2) $$WLR=\frac{outer\;diameter-lumen\;diameter}{lumen\;diameter}$$

(3) $$WCSA=\frac{\pi }{4}\times \left(outer\;diameter^{2}-lumen\;diameter^{2}\right)$$

The values measured and calculated for each of the 4 largest retinal arteries were further averaged into a single value/eye for RAOD, RALD, AWT, WLR, and WCSA.

### 2.5. Intra-Rater Agreement

**Figure 3:**
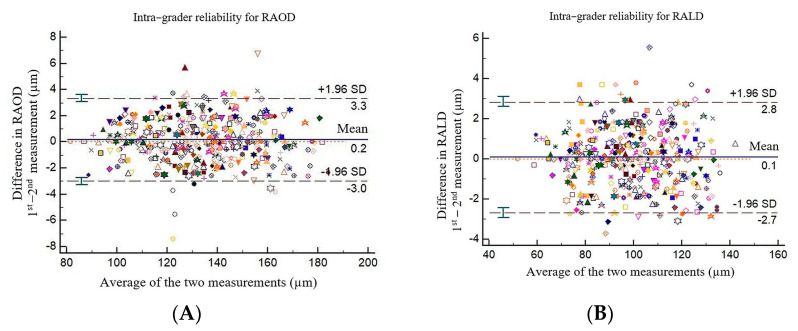
Bland–Altman plots of RAOD and RALD. (**A**) shows intra−grader reliability for RAOD and (**B**) shows intra−grader reliability for RALD. The difference was calculated by the 1st measurement minus the 2nd measurement. Pink line represents regression line of difference between 1st and 2nd measurements.

All the vessels were measured twice, one week apart, by one grader (I.D.) to compute intra-grader reliability. The intra-rater reliability was measured by the absolute agreement model of the intra-class correlation coefficient (ICC). A good agreement is indicated by a value of 0.8–1.00 (see Table 1). The mean difference between the measurements was further determined by performing Bland-Altman plot analyses using MedCalc^®^ Statistical Software version 19.5.3 (MedCalc Software Ltd., Ostend, Belgium; https://www.medcalc.org; accessed on 9 November 2020) (see Figure 3).

The coefficient of variation (%) for RAOD was 0.8991, 95% confidence interval: 0.8353–0.9628, and for RALD was: 1.0484, 95% confidence interval: 0.9740–1.1228.

### 2.6. Measurement of Ocular Factors

BCVA was measured using Snellen chart and then converted to logMAR for statistical analysis.

We used International Clinical Disease Severity Scale DR to determine the disease severity: in eyes with NDR there were no diabetic changes in the fundus examination, whereas eyes with DR had mild NPDR with the presence of a few microaneurysms, dot hemorrhages, and/or hard exudates. All fundus examinations were performed by an ophthalmologist with experience in DR.

We excluded glaucoma by assessing IOP with slit-lamp applanation tonometry doubled by analyzing RNFL and GCL thickness on OCT scans.

### 2.7. Systemic Factors

We collected demographic data, lifestyle risk factors, medical history, medication use, and also HbA1C, eGFR, and BMI values from patients’ medical records.

### 2.8. Statistical Analysis

Statistical analysis was performed using SPSS version 20.0 (SPSS, Inc., Chicago, IL, USA). Categorical data are expressed as absolute numbers while continuous data as mean ± SD (95% confidence interval) or as median (Interquartile range, IQR). The normality of data distribution was confirmed via the Kolmogorov–Smirnov test. We used parametric test: Student’s *t*-test and one-way ANOVA to compare variables with normal distribution between groups and a non-parametric Kruskal–Wallis test for variables with non-normal distribution. The Tukey test was used for post-hoc analysis to correct for multiple comparisons. Chi-square was used to compare categorical data. Univariate linear regression analyses were performed to assess associations between retinal arterial outer diameter (RAOD), retinal arterial lumen diameter (RALD), AWT, WLR, or WCSA (dependent variables) and ocular and systemic factors (independent variables). Pearson correlation analyses were performed to determine relationships between retinal morphometry variables and ocular factors. All *p*-values were two-sided, and *p*-values < 0.05 were considered significant.

## 3. Results

### 3.1. Demographic and Clinical Characteristics of the Study Sample

The demographic, systemic and ocular characteristics of the subjects are shown in Table 2. The mean age, gender, and BMI were similar between groups. 

There was a significant difference regarding the duration of DM between groups: 9.24 ± 8.10 years for NDR and 14.67 ± 6.03 years for DR (*p* = 0.006). 

Cardiovascular diseases (*p* < 0.001) and dyslipidemia (*p* < 0.001) were more frequently found in diabetic groups compared to control. 

Regarding HbA1C, blood creatinine, eGFR values and treatment with Insulin, no differences emerged between the two groups with DM (*p* > 0.05). BCVA was similar between all study groups (*p* > 0.05).

### 3.2. Morphometry of the Retinal Arteries

The parameters outlining the morphometry of retinal arteries were compared between the three groups. RAOD, RALD, AWT, and WCSA values were increased in each of the DM group versus the control but without statistical significance. There were no significant differences between NDR and DR with respect to the same parameters (see Table 3).

### 3.3. Thickness of the Retinal Layers

Peripapillary RNFL thickness was decreased in almost all quadrants within the NPDR group compared to control, the difference being statistically significant only in the nasal-inferior quadrant, 105.67 ± 22.22 µm versus 121.19 ± 21.86 µm (*p* = 0.036). Overall macular RNFL thickness was increased compared to the control, except for the inner inferior control: 25.75 ± 2.76, NDR: 24.56 ± 3.30 and NPDR: 23.29 ± 3.70 (*p* = 0.351) where it was decreased. RNFL thickness was statistically significantly increased in the inner-temporal and outer-temporal quadrants within the NPDR group compared to control (19.00 ± 2.61 versus 16.94 ± 1.05 *p* = 0.004 and 20.43 ± 2.71 versus 18.59 ± 1.19 *p* = 0.018 respectively) (see Table 4).

Regarding the GCL layer, in the NDR group all the quadrants exhibited decreased thickness compared to control, significant differences were found for inner temporal 44.67 ± 5.95 versus 47.75 ± 5.44 (*p* = 0.039), inner-nasal 47.50 ± 6.67 versus 52.34 ± 5.56 (*p* = 0.013), inner-superior 50.38 ± 6.11 versus 53.19 ± 4.82 (*p* = 0.006), and inner-inferior 48.47 ± 6.07 versus 52.97 ± 4.73 (*p* = 0.033). Except for the central thickness, center min, and center max, the NPDR group presented decreased GCL values compared to control, but without statistical significance. In the NDR group, the total retinal thickness was decreased in all quadrants, with statistical significance for inner-temporal (*p* = 0.017), inner-nasal (*p* = 0.001), inner-superior (*p* = 0.004), inner-inferior (*p* = 0.008), outer-superior (*p* = 0.038), outer nasal (*p* = 0.10), and central (*p* = 0.03) quadrants. In the NPDR group, total retinal thickness was decreased compared to control, but statistically increased compared to NDR in the central (*p* = 0.002), inner-temporal (*p* = 0.021), inner-nasal (*p* = 0.04), inner-superior (*p* = 0.035), and outer-superior (*p* = 0.033) quadrants (See Table 5).

In an univariable model (Table 6) RAOD correlated with central GCL (coefficient 0.342 per µm central GCL thickness, *p* = 0.012), RALD correlated with central GCL (coefficient 0.281 per µm central GCL, *p* = 0.041) WCSA correlated with CMT (coefficient 0.283 per µm CMT, *p* = 0.040), and central GCL (coefficient 0.353 per µm central GCL, *p* = 0.009). Retinal morphometry parameters were not associated with age, gender, duration of diabetes, HbA1c, average macular thickness, average GCL, central macular RNFL, average macular RNFL, or average peripapillary RNFL.

After we adjusted our model for age, gender, duration of diabetes and HbA1C (Table 7) WCSA correlated with the duration of diabetes (coefficient 0.311 per age duration of diabetes, *p* = 0.043), CMT (coefficient 0.316 per µm CMT, *p* = 0.029), central GCL (coefficient 0.390 per µm central GCL, *p* = 0.005). RAOD correlated with central GCL (coefficient 0.360 per µm central GCL, *p* = 0.011). RALD correlated with central GCL (coefficient 0.283 per µm central GCL, *p* = 0.050), and AWT correlated with central GCL (coefficient 0.304 per µm central GCL, *p* = 0.029). WLR correlated with average macular RNFL (coefficient 0.309 per µm increment average macular RNFL, *p* = 0.027).

Translated into clinical terms, each year increment in the duration of DM was correlated with an increase in WCSA by 0.311 µm^2^. Each µm increment of CMT was correlated with an increase in WCSA by 0.316 µm^2^, as shown also by Pearson correlation between CMT and WCSA among 62 patients with T2DM and no or minimal DR (R = 0.31, R^2^ = 0.093, *p* = 0.036). Each µm increment of central GCL was correlated with an increase in WCSA by 0.390 µm^2^ which was also emphasized by Pearson correlation (R = 0.33, R^2^ = 0.1076, *p* = 0.016). Regarding RAOD, each µm increment in central GCL was correlated with an increase in RAOD by 0.360 µm that was also validated by Pearson correlation (R = 0.33, R^2^ = 0.1094 *p* = 0.015) and each µm increment of central GCL was correlated with an increase in AWT by 0.304 µm. Each µm increment of central GCL was correlated with an increase in RALD by 0.283 and each µm increment of average macular RNFL was correlated with an increase in WLR by 0.309.

**Figure 4:**
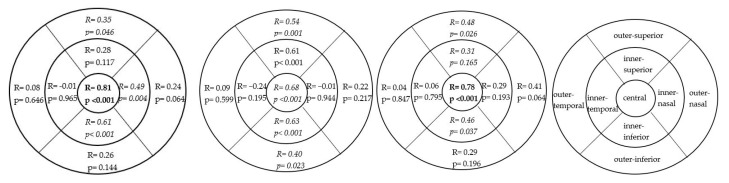
Correlation between macular RNFL and GCL thickness: left figure in control group, middle figure in NDR and right for NPDR. R, correlation coefficient; *p*, *p*-value; strong correlation is highlighted in bold and moderate correlation in italic. No significant correlation between morphometric parameters and creatinine or eGFR was found in the groups of patients with DM (*p* > 0.05).

The correlation between GCL and macular RNFL for central thickness was strongly positive in the control (R = 0.81, *p* < 0.001) and NPDR groups (R = 0.78, *p* < 0.001) and moderately positive in the NDR group (R = 0.68, *p* < 0.001). All study groups presented a moderate positive correlation in the inner-inferior (control: R = 0.61, *p* < 0.001; NDR: R = 0.68, *p* < 0.001; NPDR: R = 0.46, *p* = 0.037)and outer superior quadrants (control: R = 0.35, *p* = 0.046; NDR: R = 0.54, *p* = 0.001; NPDR: R = 0.48, *p* = 0.026) (see Figure 4).

## 4. Discussion

Progressive neurodegeneration of the retina in patients with DM in the absence of any clinically evident DR has gained attention over the last years. A 0.54 µm loss of neuroretinal thickness, meaning RNFL, GCL, and the inner plexiform layer, per year was found [20], and since neuron loss was proved to be related to deficient sensory capacity and vision-related quality of life, periodic assessments of neurodegeneration could be performed [21]. Strategies to prevent or delay the progression of DR are desired in order to decrease the economic burden associated with the care of patients with DM [21]. Studies already proved the effect of several neuroprotective factors in arresting the progression of neurodysfunction, like local administration of somatostatin in the European Consortium for the Early Treatment of Diabetic Retinopathy (EUROCONDOR) [22].

The possibility to assess retinal vessels in vivo noninvasively and directly provides researchers the opportunity to perform accurate and objective measurements of vascular caliber and further correlations with several ocular and systemic factors [15].

We did not find any significant difference between groups regarding the patients’ age and although previous studies reported narrower arteriolar diameters with increasing age, a definite correlation with age was not exhibited in our series, most probably because of the associated diseases that mask the solely age effect [23]. The similar age between groups gave us the possibility to compare diabetes-related neurodegeneration, knowing that RNFL and GCL can decrease with age in healthy individuals, as previously reported [24].

Duration of diabetes was 9.24 ± 8.10 years in the NDR group and 14.67 ± 6.03 years in the NPDR group) (*p* = 0.006) and it correlated with WCSA after adjusting for age, gender, and HbA1C: a year increment in the duration of DM predicted a 0.311 µm^2^ increase in WCSA. This observation supports the theory of vascular remodeling induced by DM, consisting of the vascular wall hypertrophy, which is reflected by an increase of the vessel wall thickness, but also of WCSA, as demonstrated by our results. In DM, hyperglycemia and oxidative stress are involved in microvascular remodeling. Exclusive control of glycemic status, although it improves microvascular function, has a modest influence on macrovascular complications. Additional macrovascular remodeling caused by high blood pressure should be considered. In type 2 DM concomitant hypertension was found to induce inward remodeling of small arteries and attenuation of vessel dilation, which could partially explain the small differences between the control group and DM groups in our study [25]. Previous studies noted increased RAOD and AWT in patients with DM [26,27,28,29,30,31,32] compared to Wong et al. [33] who found a decreased retinal arteriolar diameter to be associated with the risk of diabetes and coronary artery disease. We found similar WLR in the DM groups as compared to control, contrary to other studies in which WLR was increased in patients with DM [34]. We should clearly emphasize that the coexistence of hypertension in most of the patients with DM is limiting the analysis of a pure impact of DM on the vessels since high blood pressure also has a strong impact on arterial remodeling. Retinal vessel structure is of utmost importance because any deviation from the optimum design principle that allows sufficient blood distribution with the least amount of energy, will cause less efficient circulation and interrupt the metabolic capacity, especially in DM [34]. Furthermore, we did not find a correlation between increased RAOD and nephropathy as other studies did [28].

Cardiovascular diseases and dyslipidemia were frequently associated in the DM groups as compared to control in our series, but no difference emerged between the NDR and NPDR groups.

As previously described, retinal neurons are organized into three layers of nerve cell bodies and two layers of synapses, as follows: the outer nuclear layer contains rod and cone photoreceptor cells, the inner nuclear layer, cell bodies of second and third-order neurons, especially bipolar, horizontal, and amacrine cells, and the ganglion cell layer is formed by the cell bodies of ganglion cells, displaced amacrine cells, and astrocytes [13]. Hombrebueno et al. state that although most of the retinal neurons are affected in DM, those situated in the inner retina, such as ganglion and amacrine cells, presented more severe degeneration compared to neuronal cells located closer to the outer retina [35]. One possible explanation is the increased susceptibility to hyperglycemia manifested by the inner retinal neurons, which makes them more fragile to changes regarding neurotrophic factors [35]. Another explanation could be that VEGF depletion causes inner blood retinal barrier (BRB) damage that induces vascular dysfunction-dependent neurodegeneration [35,36]. In our series we found significantly decreased GCL thickness in the inner quadrants of NDR eyes compared to the control group, which is similar to other findings [13,30,37,38,39,40]. The NPDR group also exhibited decreased GCL thickness compared to the control, but the differences were not statistically significant. This could be due to the dual presence of neurodegeneration that induces thinning and incipient macular edema that translates into thickening, as explained by Moran et al. [13]. We found an argument to support this theory when we analyzed the total macular thickness, which was increased in the NPDR group compared to the control, but also to NDR. In the NDR group we found a decreased total retinal thickness that was attributed by other authors mainly to the thinning of GCL and RNFL [40].

In the NPDR group, macular edema was not apparent clinically or on OCT. However, upon analyzing the OCT scans while processing the images, we observed either a discrete diffuse thickening or small cysts in the OPL or INL layers. These findings led us to the conclusion that the increased macular volume in NPDR may be attributed to incipient macular edema.

Regarding the peripapillary RNFL thickness, the results were not so obvious. We found a decreased RNFL thickness only in the inner-nasal quadrant within the NPDR group: 105.67 ± 22.22 µm compared to the control 121.19 ± 21.86 µm *p* = 0.036, while, for macular RNFL, we found increased RNFL thickness in the NPDR group in the outer-temporal (19.00 ± 2.61) and inner-temporal quadrant (20.43 ± 2.71) compared to the control (16.94 ± 1.05 and 18.59 ± 1.19) (*p* = 0.004 and *p* = 0.018). It seems more appropriate to analyze peripapillary RNFL because the thickness is normally increased and it is easier to detect subtle changes. Therefore, we will further focus on the decreased value found in the peripapillary location. Shahidi et al. [41] found that inferior decreased RNFL was significantly associated with peripheral neuropathy in patients with type 2 DM. We did not look for this association, but it brings new perspectives regarding the pathogenetic mechanisms of DR.

Linear regression provided us important insights regarding arterial parameters and neurodegeneration. We found a significant positive correlation between RAOD, RALD, AWT, WCSA, and central GCL thickness in early stages of DR, as previously reported [1] which was preserved after we adjusted for age, gender, duration of diabetes, and HbA1C. An interesting finding was the positive correlation between the duration of DM and WCSA, which is another argument in favor of the vascular remodeling. Previous studies found significant correlations between structural and functional neurodegeneration and increased venous diameter in adolescents with type 2 DM [42] or between the amplitude and implicit times of rod-derived ERG response and retinal arteriolar caliber [43]. WLR was correlated with average macular RNFL. The correlation we found between almost all analyzed arterial parameters, except for WLR and central GCL, confirms the differences we found when we compared retinal layers thickness. We could further speculate that ganglion cells seem to be the first ones affected in no or mild DR and that there is a subtle interplay between neurodegeneration and retinal arteries in the initial stages of DR.

Analyzing more deeply, researchers found mildly increased arterial StO_2_ (oxygen saturation), decreased venous StO_2_, and increased mean arterio-venous (A–V) difference in NPDR patients [44]. Therefore, the authors suggested that the mildly hypoxic retina is characterized by an increased extraction of oxygen which consequently leads to a lower venous StO_2_ and a higher A–V difference. The increased oxygen extraction represents either increased oxygen consumption by the retinal tissue, or a compensatory mechanism in response to the reduction of blood flow in areas with retinal hypoxia [44]. This observation opens new perspectives when studying early retinal changes in patients with DM.

The strengths of our study are: it is prospective; the type 2 DM groups included patients with no or mild DR allowing us to better analyze the changes related to neurodegeneration; we adjusted our model for the effect of confounding factors before confirming the correlations between arterial parameters and structural neurodegeneration; we included the largest four arteries at 0.5 DD from the optic nerve, not just the superior-temporal artery. Our study has some limitations. First, the sample size is relatively small; a larger number of patients included in each group would have increased the strength of the study. Second, we used a manual method to measure outer and luminal arterial diameter which although proved to be accurate, and it is time consuming compared to an automatized software. Third, we included larger arteries instead of arterioles, which could be affected before larger vessels. Forth, to increase the precision of the measurement, adaptive optics scanning laser ophthalmoscope (AOSLO) technique would have enabled us to delimit more accurately the retinal vessel from the background and to measure the luminal diameter. Fifth, we did not include in our analysis the axial length (AL) or disc area (DA), which were proven to have a strong effect on RNFL thickness in non-glaucomatous eyes [45,46]. As AL increases, RNFL decreases and a smaller DA is related to a thinner RNFL. Sixth, we did not include in our analysis the optic disc size and axial length, which could have given us more accurate RNFL measurements.

To the best of our knowledge, this approach of analyzing multiple parameters of retinal arteries, not just the outer diameter, and neurodegeneration has not been applied before using the FWHM method.

## 5. Conclusions

Inner GCL thickness was decreased in patients with DM, while the morphometric parameters of the retinal arteries were similar between groups. RAOD, RALD, AWT, and WCSA were associated with the central GCL thickness in patients with no or mild DR, opening new perspectives in the understanding of DR.

## Figures and Tables

**Table 1:** Intra-grader reliability assessment of outer diameter and lumen diameter in 85 subjects.

ICC for RAOD	ICC	95% CI	ICC for RALD	ICC	95% CI
For single measures	0.9966	0.9958–0.9972	for single measures	0.9966	0.9959–0.9972
For average measures	0.9983	0.9979–0.9986	for average measures	0.9983	0.9979–0.9986

ICC, intraclass correlation coefficient; CI, confidence interval; RAOD; retinal artery outer diameter; RALD; retinal artery lumen diameter.

**Table 2:** Demographic and clinical characteristics of the study sample.

Characteristics	Control	NDR	DR	*p*-Value
Number of subjects	32	32	21	
Age, years	55.34 ± 12.37 (51.054, 59.626)	60.50 ± 9.95 (57.053, 63.947)	60.48 ± 9.53 (56.404, 64.556)	0.112 ^^^
Gender: Male/Female, n	17/15	18/14	15/6	0.388 ^#^
BMI, kg/m^2^	27.16 ± 5.03 (25.417, 28.903)	32.12 ± 7.23 (29.615, 34.625)	29.44 ± 4.84 (27.369, 31.510)	**0.005 ^^^**
Diabetes duration, years	N/A	9.24 ± 8.10 (6.433, 12.046)	14.67 ± 6.0 (12.091, 17.249)	**0.006 ^˅^**
HbA1c, %	N/A	9.25 ± 2.30 (8.453, 10.047)	9.28 ± 1.79 (8.514, 10.046)	0.397 ^˅^
Blood creatinine, mmol/L	N/A	0.83 ± 0.21 (0.757, 0.903)	1.05 ± 0.53 (0.823, 1.277)	0.849 ^˅^
eGFR, mL/min/1.73 m^2^	N/A	88.19 ± 20.71 (81.014, 95.365)	74.25 ± 28.61 (62.014, 86.486)	0.136 ^˅^
Current smoking, n	8	10	8	0.596 ^#^
BCVA, logMAR	0.00 (0)	0.00 (0.1)	0.00 (0.1)	0.185 *
Treatment with Insulin, n	N/A	21	17	0.125 ^#^
HBP, n	17	24	13	0.189 ^#^
Cardiovascular disease, n	3	14	12	**<0.001 ^#^**
Dyslipidemia, n	6	18	16	**<0.001 ^#^**

Data are expressed as mean ± standard deviation (95% confidence interval) or as median (interquartile range), unless otherwise indicated. Statistically significant *p* values are highlighted as bold. ^^^ One-way ANOVA test, * Kruskal–Wallis test, ^#^ Chi-square test, ^˅^ *t*-test for independent means; n, number; NDR, no diabetic retinopathy; DR, diabetic retinopathy BMI, body mass index; HbA1c, glycated hemoglobin; eGFR, estimated glomerular filtration rate; BCVA, Best corrected visual acuity; LogMAR, logarithm of the minimum angle of resolution. HBP, high blood pressure.

**Table 3:** Morphometry of the retinal arteries.

	Control	NDR	NPDR	*p*-Value
RAOD (µm)	130.71 ± 10.08 (127.217, 134.203)	133.73 ± 8.44 (130.806, 136.654)	135.76 ± 10.04 (131.466, 140.054)	0.155 ^^^
RALD (µm)	97.21 ± 8.10 (94.404, 100.016)	98.78 ± 6.87 (96.399, 101.160)	100.38 ± 8.12 (96.907, 103.853)	0.338 ^^^
AWT (µm)	16.75 ± 2.19 (15.991, 17.509)	17.47 ± 1.90 (16.812, 18.128)	17.69 ± 2.13 (16.779, 18.601)	0.211 ^^^
WLR	0.35 ± 0.05 (0.333, 0.367)	0.36 ± 0.04 (0.346, 0.374)	0.35 ± 0.05 (0.329, 0.371)	0.675 ^^^
WCSA (µm^2^)	6065.36 ± 1162.26 (5662.555, 6467.945)	6391.15 ± 977.05 (6052.626, 6729.674)	6646.89 ± 1085.16 (6182.768, 7111.012)	0.151 ^^^

Data are expressed as mean ± standard deviation (95% confidence interval), unless otherwise indicated. ^^^ One-way ANOVA test; NDR, no diabetic retinopathy; NPDR, diabetic retinopathy; RAOD, retinal artery outer diameter; RALD, retinal artery lumen diameter; AWT, arterial wall thickness; WLR, wall-to-lumen ratio; WCSA, wall cross-sectional area.

**Table 4:** RNFL thickness among groups.

Retinal Layer Thickness (µm)	Control	NDR	NPDR	*p*-Value	Post-Hoc Analysis with Tukey Test		
*peripapillary RNFL*					**Control-NDR**	**Control-NPDR**	**NDR-NPDR**
Temporal	71.41 ± 12.90 (66.941, 75.879)	68.22 ± 12.5 (63.868, 72.572)	73.29 ± 12.78 (67.952, 78.628)	0.342 ^^^			
Temporal-superior	142.16 ± 20.02 (135.224, 149.096)	135.63 ± 22.38 (127.876, 143.384)	138.62 ± 21.28 (129.519, 147.721)	0.472 ^^^			
Nasal-superior	116.38 ± 14.20 (111.46, 121.3)	115.78 ± 22.32 (108.047, 123.513)	113.95 ± 23.01 (104.109, 123.791)	0.907 ^^^			
Nasal	78.88 ± 12.97 (74.386, 83.374)	74.97 ± 13.97 (70.129, 79.810)	72.76 ± 1322 (67.106, 78.414)	0.243 ^^^			
Nasal-inferior	121.19 ± 21.86 (113.616, 128.764)	113.88 ± 23.89 (105.603, 122.157)	105.67 ± 22.22 (96.166, 115.174)	0.056 ^^^	0.464	**0.036**	0.381
Temporal-inferior	144.47 ± 17.36 (108.455, 120.485)	142.28 ± 20.22 (135.274, 149.286)	137.05 ± 26.62 (125.665, 148.435)	0.451 ^^^			
Average	103.19 ± 17.47 (97.044, 109.336)	99.19 ± 10.28 (95.628, 102.752)	98.48 ± 11.61 (93.514, 103.446)	0.168 ^^^			
*macular RNFL*							
Central	12.50 ± 1.97 (11.817, 13.183)	12.44 ± 2.18 (11.685, 13.195)	13.57 ± 2.98 (12.295, 14.845)	0.176 ^^^			
Center min	0 (1)	0 (0)	0 (2)	0.224 *			
Center max	23.19 ± 2.63 (22.279, 24.101)	23.75 ± 3.20 (22.641, 24.859)	23.81 ± 3.71 (22.926, 25.834)	0.706 ^^^			
Inner-temporal	16.94 ± 1.05 (16.576, 17.304)	17.59 ± 1.70 (17.001, 18.179)	18.67 ± 3.02 (17.88, 20.12)	**0.008 ^^^**	0.425	**0.004**	0.107
Inner-nasal	21.09 ± 1.67 (20.511, 21.669)	21.66 ± 4.35 (20.153, 23.167)	22.71 ± 4.44 (21.707, 24.872)	0.283 ^^^			
Inner-superior	24.44 ± 2.80 (21.469, 23.410)	23.97 ± 3.15 (22.879, 25.061)	26.29 ± 6.08 (24.602, 29.118)	0.107 ^^^			
Inner-inferior	25.75 ± 2.76 (24.794, 26.706)	24.56 ± 3.30 (23.417, 25.703)	26.05 ± 6.26 (21.708, 24.872)	0.351 ^^^			
Outer-temporal	18.59 ± 1.19 (18.178, 19.002)	19.00 ± 1.48 (18.49, 19.51)	20.10 ± 3.27 (18.701, 21.499)	**0.029 ^^^**	0.733	**0.018**	0.112
Outer-nasal	49.19 ± 6.37 (46.983, 51.397)	45.59 ± 6.49 (43.341, 47.839)	47.10 ± 11.48 (42.19, 52.01)	0.201 ^^^			
Outer-superior	37.25 ± 5.36	35.34 ± 5.31	38.19 ± 8.12	0.224 ^^^			
Outer-inferior	39.22 ± 4.34 (37.716, 40.724)	38.16 ± 6.25 (35.994, 40.325)	39.14 ± 9.30 (35.162, 43.118)	0.781 ^^^			
Average	27.22 ± 1.97 (26.537, 27.903)	26.48 ± 2.58 (25.586, 27.374)	27.98 ± 5.19 (25.760, 30.199)	0.259 ^^^			

Data are expressed as mean ± standard deviation (95% confidence interval) or as median (Interquartile range) unless otherwise indicated. Statistically significant *p* values are highlighted as bold. ^^^ One-way ANOVA test * Kruskal–Wallis test; NDR, no diabetic retinopathy; NPDR, non-proliferative diabetic retinopathy; RNFL, retinal nerve fiber layer.

**Table 5:** GCL and macular thickness among groups.

***Retinal Layer Thickness* (µm)**	**Control**	**NDR**	**NPDR**	*p*-Value *	**Post-Hoc Analysis with Tukey Test**		
***GCL***					**Control-NDR**	**Control-NPDR**	**NDR-NPDR**
Central	16.16 ± 4.68 (14.498, 17.741)	15.06 ± 4.11 (13.636, 16.484)	17.81 ± 7.70 (14.517, 21.103)	0.199 ^^^			
Center min	0.5(2)	1(2)	0(1.5)	0.594 *			
Center max	38.81 ± 8.94 (35.712, 41.907)	36.72 ± 9.74 (33.328, 40.112)	39.33 ± 9.68 (35.189, 43.470)	0.544 ^^^			
Inner-temporal	47.75 ± 5.44 (45.865, 49.635)	43.59 ± 6.94 (41.185, 45.994)	44.67 ± 5.95 (42.125, 47.215)	**0.025 ^^^**	**0.039**	0.161	0.797
Inner-nasal	52.34 ± 5.56 (50.414, 54.266)	47.50 ± 6.67 (45.189, 49.811)	49.86 ± 6.32 (47.157, 52.563)	**0.009 ^^^**	**0.013**	0.304	0.342
Inner-superior	53.19 ± 4.82 (51.52, 54.86)	48.47 ± 5.61 (46.526, 50.414)	50.38 ± 6.11 (47.767, 52.993)	**0.004 ^^^**	**0.006**	0.146	0.403
Inner-inferior	52.97 ± 4.73 (51.331, 54.609)	48.47 ± 6.07 (46.367, 50.573)	48.81 ± 8.98 (44.969, 52.651)	**0.013 ^^^**	**0.033**	0.052	0.979
Outer-temporal	36.50 ± 3.70 (35.218, 37.782)	35.22 ± 4.96 (33.501, 36.938)	35.33 ± 5.58 (32.943, 37.717)	0.502 ^^^			
Outer-nasal	38.53 ± 3.11 (37.452, 39.607)	37.66 ± 3.53 (36.437, 38.883)	36.24 ± 4.33 (34.388, 38.092)	0.082 ^^^			
Outer-superior	34.81 ± 3.49 (33.601, 36.019)	33.72 ± 3.25 (32.594, 34.846)	33.48 ± 4.32 (31.632, 35.328)	0.336 ^^^			
Outer-inferior	33.56 ± 2.59 (32.663, 34.457)	32.72 ± 3.59 (31.476, 33.964)	32.62 ± 3.96 (30.926, 34.314)	0.500 ^^^			
Average	40.65 ± 3.29 (39.510, 41.789)	38.05 ± 3.95 (36.681, 39.419)	38.80 ± 4.19 (37.008, 40.592)	**0.023 ^^^**	**0.031**	0.229	0.626
	**Control**	**NDR**	**NPDR**	***p*-Value**	**Post-Hoc Analysis with Tukey Test**		
*Total retinal thickness*					**Control-NDR**	**Control-NPDR**	**NDR-NPDR**
Central	280.19 ± 21.38 (272.782, 287.598)	264.44 ± 22.20 (256.748, 272.132)	286.19 ± 24.07 (275.895, 296.485)	**0.002 ^^^**	**0.03**	0.585	**0.002**
Center min	230.47 ± 19.81 (223.606, 237.334)	212.97 ± 21.28 (205.597, 220.343)	230.71 ± 27.96 (218.751,242.668)	**0.003 ^^^**	**0.015**	0.999	**0.013**
Center max	326.84 ± 19.81 (319.976, 333.704)	314.69 ± 22.92 (306.749, 322.631)	332.43 ± 23.98 (322.174, 342.686)	**0.016 ^^^**	0.128	0.641	**0.015**
Inner-temporal	332.13 ± 16.38 (326.455, 337.805)	318.44 ± 18.30 (312.099, 324.780)	331.71 ± 19.90 (323.199, 340.221)	**0.005 ^^^**	**0.017**	0.996	**0.021**
Inner-nasal	348.94 ± 16.24 (343.313, 354.567)	330.88 ± 21.44 (323.452, 338.308)	347.43 ± 16.27 (340.471, 354.389)	**<0.001 ^^^**	**0.001**	0.951	**0.004**
Inner-superior	346.41 ± 16.00 (340.866, 351.954)	330.75 ± 17.07 (324.836, 336.664)	342.76 ± 20.31 (334.073, 351.447)	**0.002 ^^^**	**0.004**	0.724	**0.035**
Inner-inferior	343.56 ± 15.53 (338.179, 348.941)	328.53 ± 17.48 (322.474, 334.586)	339.52 ± 22.04 (330.093, 348.946)	**0.004 ^^^**	**0.008**	0.687	0.068
Outer-temporal	286.38 ± 10.95 (282.586, 290.174)	278.41 ± 15.24 (273.129, 283.690)	287.90 ± 19.08 (279.739, 296.061)	**0.039 ^^^**	0.125	0.924	0.054
Outer-nasal	316.28 ± 13.13 (311.731, 320.829)	304.66 ± 15.15 (299.411, 309.909)	314.19 ± 24.17 (303.852, 324.527)	**0.021 ^^^**	**0.038**	0.895	0.107
Outer-superior	300.66 ± 12.66 (296.274, 305.046)	288.78 ± 12.15 (284.570, 292.989)	298.95 ± 20.16 (290.328, 307.572)	**0.004 ^^^**	**0.010**	0.904	**0.033**
Outer-inferior	288.38 ± 11.58 (284.368, 292.392)	280.84 ± 17.60 (274.742, 286.938)	289.19 ± 17.02 (281.911, 296.469)	0.081 ^^^			
Average	315.88 ± 12.48 (311.556, 320.204)	302.66 ± 14.07 (297.785, 307.535)	315.32 ± 16.79 (308.139, 322.501)	**<0.001 ^^^**	**0.003**	0.988	**0.004**

Data are expressed as mean ± standard deviation (95% confidence interval), or as median (interquartile range) unless otherwise indicated. Statistically significant *p* values are highlighted as bold. ^^^ One-way ANOVA test, * Kruskal–Wallis Test. NDR, no diabetic retinopathy; NPDR, diabetic retinopathy; GCL, ganglion cell layer.

**Table 6:** Linear regression univariate analysis between systemic and ocular factors associated with retinal artery morphometry parameters.

	RAOD	RALD	AWT	WLR	WCSA
	Univariable Model	Univariable Model	Univariable Model	Univariable Model	Univariable Model
Age, years	0.004 (−0.259, 0.267) (0.976)	−0.065 (−0.278, 0.148) (0.645)	0.130 (0.074, 0.186) (0.353)	0.142 (0.140, 0.144) (0.310)	0.113 (−29.322, 29.548) (0.422)
Gender, male	0.061 (−5.141, 5.263) (0.664)	0.126 (−4.066, 4.318) (0.370)	−0.093 (−1.225, 1.039) (0.506)	−0.197 (−0.221, −0.173) (0.158)	−0.052 (−584.519, 584.416) (0.712)
Duration of diabetes, years	0.205 (−0.116, 0.526) (0.141)	0.134 (−0.131, 0.399) (0.339)	0.220 (0.150, 0.290) (0.113)	0.110 (0.108, 0.112) (0.433)	0.265 (−35.323, 35.853) (0.055)
HbA1C, %	−0.074 (−1.293, 1.145) (0.602)	−0.139 (−1.117, 0.839) (0.326)	0.089 (−0.174, 0.352) (0.531)	0.177 (0.171, 0.183) (0.209)	0.003 (−136.675, 136.681) (0.981)
Centralmacular thickness (µm)	0.270 (0.172, 0.368) (0.051)	0.228 (0.148, 0.308) (0.100)	0.194 (0.172, 0.216) (0.163)	0.041 (0.041, 0.0412) (0.773)	0.283 (−10.644, 11.210) **(0.040)**
Macular thickness average (µm)	0.126 (−0.029, 0.281) (0.368)	0.121 (−0.005, 0.247) (0.387)	0.064 (0.030, 0.098) (0.649)	−0.013 (−0.0128, −0.013) (0.925)	0.106 (−17.374, 17.586) (0.450)
Central GCL (µm)	0.342 (−0.055, 0.739) **(0.012)**	0.281 (−0.048, 0.610) **(0.041)**	0.260 (0.172, 0.348) (0.060)	0.074 (0.072, 0.076) (0.597)	0.353 (−44.112, 44.817) **(0.009)**
Average GCL (µm)	0.228 (−0.388, 0.844) (0.101)	0.241 (−0.257, 0.739) (0.082)	0.075 (−0.063, 0.213) (0.594)	−0.082 (−0.086, −0.078) (0.560)	0.156 (−70.137, 70.449) (0.266)
Central macular RNFL, (µm)	0.192 (−0.786, 1.170) (0.168)	0.177 (−0.618, 0.972) (0.204)	0.111 (−0.106, 0.328) (0.428)	−0.014 (−0.018, −0.009) (0.920)	0.181 (−109.760, 110.122) (0.194)
Average macular RNFL, (µm)	−0.027 (−0.687, 0.633) (0.850)	−0.110 (−0.422, 0.642) (0.432)	0.144 (0.001, 0.286) (0.304)	0.194 (0.189, 0.198) (0.164)	−0.015 (−74.209, 74.179) (0.916)
Average peripapillary RNFL (µm)	0.183 (−0.050, 0.416) (0.190)	0.163 (−0.028, 0.354) (0.243)	0.116 (0.064, 0.168) (0.408)	0.002 (−0.00001, 0.004) (0.988)	0.141 (−26.295, 26.577) (0.315)

Statistically significant *p* values are highlighted as bold. Figures in parenthesis from middle row indicate CI for beta coefficient. Figures in parenthesis from lower row indicate *p* values; *p* < 0.05 was considered statistically significantly different. RAOD, retinal artery outer diameter; RALD, retinal artery lumen diameter; AWT, arterial wall thickness; WLR, wall-to-lumen ratio; WCSA, wall cross-sectional area; GCL, ganglion cell later; RNFL, retinal nerve fiber layer.

**Table 7:** Linear regression analysis using an adjusted model.

	RAOD	RALD	AWT	WLR	WCSA
	Adjusted Model *	Adjusted Model *	Adjusted Model *	Adjusted Model *	Adjusted Model *
Age, years	−0.065 (−0.351, 0.221) (0.666)	−0.119 (−0.348, 0.110) (0.433)	0.071 (0.011, 0.131) (0.628)	0.124 (0.122, 0.126) (0.394)	0.032 (−30.969, 31.033) (0.827)
Gender, male	−0.014 (−5.458, 5.486) (0.922)	0.086 (−4.332, 4.504) (0.556)	−0.128 (−1.275, 1.019) (0.369)	−0.207 (−0.231, −0.183) (0.146)	−0.103 (−595.876, 595.669) (0.473)
Duration of diabetes, years	0.214 (−0.152, 0.580) (0.170)	0.111 (−0.185, 0.407) (0.471)	0.287 (0.210, 0.363) (0.061)	0.196 (0.194, 0.198) (0.191)	0.311 (−39.497, 40.119) **(0.043)**
HbA1C, %	−0.042 (−1.319, 1.235) (0.775)	−0.134 (−1.166, 0.898) (0.369)	0.152 (−0.115, 0.419) (0.292)	0.227 (0.221, 0.233) (0.116)	0.066 (−138.999, 139.132) (0.648)
Central macular thickness, µm	−0.263 (0.152, 0.374) (0.078)	0.193 (0.102, 0.284) (0.199)	0.248 (0.226, 0.270) (0.088)	0.120 (0.119, 0.1202) (0.408)	0.316 (−11.437, 12.069) **(0.029)**
Macular thickness average, µm	0.107 (−0.064, 0.278) (0.469)	0.070 (−0.069, 0.209) (0.637)	0.117 (0.081, 0.153) (0.412)	0.067 (0.0668, 0.0672) (0.636)	0.136 (−18.399, 18.671) (0.343)
Central GCL, µm	0.360 (−0.055, 0.775) **(0.011)**	0.283 (−0.061, 0.627) **(0.050)**	0.304 (0.215, 0.393) **(0.029)**	0.118 (0.116, 0.120) (0.403)	0.390 (−44.041, 44.821) **(0.005)**
Average GCL, µm	0.273 (−0.442, 0.988) (0.073)	0.258 (−0.322, 0.838) (0.091)	0.149 (−0.004, 0.302) (0.319)	−0.043 (−0.047, −0.039) (0.772)	0.229 (−78.256, 78.714) (0.126)
Central macular RNFL, µm	0.180 (−0.882, 1.259) (0.207)	0.123 (−0.751, 0.997) (0.416)	0.209 (−0.014, 0.432) (0.150)	0.113 (0.109, 0.117) (0.434)	0.247 (−114.687, 115.181) (0.089)
Average macular RNFL, µm	0.009 (−0.722, 0.739) (0.952)	−0.129 (−0.715, 0.457) (0.386)	0.263 (0.116, 0.409) (0.065)	0.309 (0.305, 0.313) **(0.027)**	0.077 (−79.303, 79.457) (0.595)
Average peripapillary RNFL, µm	0.247 (−0.011, 0.505) (0.108)	0.201 (−0.008, 0.410) (0.192)	0.196 (0.142, 0.250) (0.191)	0.036 (0.034, 0.038) (0.809)	0.227 (−27.897, 28.351) (0.130)

Statistically significant *p* values are highlighted as bold. Figures in parenthesis from middle row indicate CI for beta coefficient. Figures in parenthesis from lower row indicate *p* values; *p* < 0.05 was considered statistically significantly different. RAOD, retinal artery outer diameter; RALD, retinal artery lumen diameter; AWT, arterial wall thickness; WLR, wall-to-lumen ratio; WCSA, wall cross-sectional area; GCL, ganglion cell later; RNFL, retinal nerve fiber layer. * adjusted for age, gender, duration of diabetes, and HbA1C.

## Data Availability

The data presented in this study are available on request from the corresponding author.
